# Establishing an environment in which rigorous scientific inquiry is practiced: a personal journey

**DOI:** 10.1093/nar/gkac526

**Published:** 2022-07-08

**Authors:** Stanley T Crooke

**Affiliations:** n-Lorem Foundation, Carlsbad, CA, USA

## Abstract

For more than three decades, Ionis Pharmaceutics has pursued the challenging mission of creating a new platform for drug discovery. To overcome the numerous challenges faced required the integration of innovation across many scientific areas, despite many disappointments and failures. The approaches implemented to create and maintain a scientific environment to achieve the mission demanded the rigorous practice of science over three decades. The approaches taken are discussed in this perspective.

## INTRODUCTION

What is rigorous science? I believe that I have always submitted manuscripts that represented thorough, reproducible experiments from which only conclusions supported by the data were drawn. However, like every other scientist I have known, more frequently than I would like, referees have suggested added experiments and sometimes added controls that were needed. Sometimes those referee comments added value well beyond the usual dotting Is and T crossing exercise that often enhances the quality and readability of a manuscript. Did those manuscripts reflect inadequate commitment to rigorous science? I have also published a manuscript that stated one conclusion (1), then published another paper that came to a diametrically opposite conclusion (2). Did those papers represent inadequate or incompetent science? In my opinion, no. In fact, I think they represent the best practice of the scientific method. At the time of the first publication, the preponderance of data supported the conclusion (3). However, as follow up experiments were performed and other studies using different methods advanced, it became clear that numerous observations did not support the initial conclusion and as a result, we continued to investigate and followed the data to the inescapable conclusion that our earlier interpretation of the data was wrong. We published the studies that convinced us that the earlier conclusions were incorrect and the work that led to the promulgation of a new step by step molecular mechanism became one of the most important publications in the field (2).

Even more daunting than defining rigorous science, however, are the deeper, more important questions. How do rigorous scientific processes relate to the ethical practice of science? How does the ethical practice of science relate to the ethics of individuals? Is the scientific method simply a codification of the search for the truth and the expectation that the truth will be told? What are the inducements to engage in these challenging personal endeavors that all of us know are far too often ignored in the practice of life by others and, disappointingly, by leaders and heroes? What are the pressures that drive reasonable people who have invested a decade or more in advanced training to become a scientist, only to abandon well understood best practices and even lie about or fabricate data? Then there are more practical questions such as how do the risks and impacts of inadequate rigor or misconduct vary in regard to basic vs therapeutic research or compared to clinical research? How do the pressures on academic scientists differ from those experienced by scientists in industry? Finally, how is an ethical, rigorous scientific environment fostered and sustained?

The specific issues germane to science are intertwined with the practical personal considerations with which most of us must contend every day, such as how do I get ahead, how am I to achieve peer recognition and with ethical and moral considerations that have occupied philosophers throughout human history. I would not suggest that I am wise enough to provide an applicable solution or a prescription for others in these weighty matters. I also know that addressing the simpler elements in an enormously complex equation is insufficient to address the important issues that must be discussed if meaningful progress is to be achieved.

How does one foster an environment in which rigorous scientific enquiry is the norm? I will describe my journey in basic and therapeutic research and how I have tried to meet my responsibilities as scientist and a physician for others to consider.

A great deal of attention has been invested in enhancing the rigor and precision of science and in understanding the factors that may enhance the quality of science and the pressures that may conduce to sloppy or unethical science. These efforts have led to numerous recommendations, a few of which are practical and have been adopted. Because these efforts have been discussed extensively in the literature, I will not review them here, but do acknowledge the important impact on my thinking about this manuscript, so I have included a few references that were particularly influential as I prepared this perspective (4–11).

### Pressures that are unique to corporate science and the implications of mistakes as a function of the focus of research activities

Science is the same whether practiced in academia or industry, but there are several types of demands uniquely experienced by scientists in the industry that can affect behavior. The simplest pressure to understand is conflicting regulatory demands experienced by publicly traded companies. Securities regulations require that material events be disclosed promptly in a fashion that assures all investors have equal access. What event or data may be material is often not clear and decided post-facto by whether the stock price shifts. Thus, quite often, top line or preliminary data must be disclosed publicly, while detailed analyses to understand why the results are as they are may take months.

Even more problematic are situations in which the sponsor company is in confidential discussions with regulatory authorities. The compromises made to comply with various constituencies and regulatory agencies can often be misunderstood. More complicated issues derive from the fact that most drugs that enter development fail. As a general rule, most therapeutic agents in development experience challenges that could derail development and industry leaders know that for drugs to overcome these hurdles, the leaders must be strong champions for the drug. The challenge here is to select leaders who can balance being a champion with being a rigorous scientist committed discovering the truth within the limits of analytical tools that are available.

As the focus of scientific enquiries shifts from basic biological research to biological research with therapeutic purpose and then to clinical research, the stakes become higher because the impacts of mistakes are greater. If applied therapeutic research is poorly performed, opportunities to advance new treatments may be lost.

Of even greater concern is the possibility that inadequate scientific efforts may lead to the advancement of potentially toxic or ineffective drugs to clinical trials. Clinical trials and commercial development of a drug directly expose other humans to risks, some of which may be predicted, while many others are simply not known or predictable. Thus, the lives of test subjects can be directly affected positively or negatively, the lives of patients with a disease that might be treated with a drug may be altered and the practice of medicine may change in response to the results of a clinical trial. The futures of companies, the lives of hundreds to thousands of employees and the fortunes of many investors can be affected.

These factors, then, place an even greater premium on the rigorous pursuit of science, integrity, and judgement. The potential impacts of these risks and the management of risks have a profound effect on the practice of science in the drug discovery and development industry that are difficult to fully appreciate unless experienced personally.

## CREATING A CULTURE IN WHICH THE ETHICAL AND RIGOROUS PRACTICE OF SCIENCE IS THE NORM

I have now had a long career during which I was privileged to hold senior leadership positions in the drug discovery and development industry while simultaneously enjoying very active positions in academic medicine and science. I have been a hands-on scientific leader throughout my career. In fact, I am shocked to say that I think that today I am doing the most innovative and important science in my career at an age that I once thought was old. My career has thus given me the opportunity to learn from many experiences and a number of these events occurred early enough in my career to be formative.

Critically, the first 15 years or so of my career were spent in two very different large pharmaceutical companies, Bristol-Myers (now BMS) and Smith Kline Beckman (now GSK). I took many critical insights from both companies. Some insights were related to systems and approaches that I thought encouraged rigorous, innovative science. Other observations involved events that were extremely detrimental. Positive or negative, I applied these lessons to the plan that I created when I founded Ionis.

### Establishing and maintaining a rigorous scientific environment at Ionis

In 1989, essentially nothing was known about RNA targeted drug (RTD) discovery, creating a formidable mission, which was the creation from scratch, of a new drug discovery platform. On the other hand, I saw the task as representing the extraordinary opportunity of taking a blank piece of paper and filling it with progressively more detail as the technology was created and advanced.

To meet these challenges, a new organization with unique skills was required. A new medicinal chemistry, the medicinal chemistry of oligonucleotides and their efficient synthesis, had to be established and coupled to the in depth understanding of nucleic acid biochemistry, the molecular and cellular biology of RNA, molecular pharmacology and drug discovery and development expertise. A conceptual framework to support thinking of RNAs as a series of receptor sites for oligonucleotides was essential, as was understanding the pharmacokinetics and toxicological properties of chemically modified oligonucleotides. The only relevant experience related to therapeutic oligonucleotides derived from work in the 1970s using oligonucleotides such as poly-rI:rC (12). I concluded that a unique, innovation-centered organization would be needed to support the decades long effort that lay before us.

### Culture

Culture is a word that is widely used to describe nebulous qualities of environments and organizations but is difficult to define operationally. The way I prefer to think of a culture is that it is a fabric on which all the aspirations, people, systems and activities of an organization reside. The fabric is comprised of the core ethics and beliefs of the organization and the people and systems that comprise that institution. In strong, effective organizations, the ethics and beliefs expressed in the culture statement on the wall are manifested every day by the behaviors of the leaders of the organization and the types of behaviors that are rewarded.

In strong organizations, all are aligned with the key tasks and aspirations, and the system supports the aspirations, beliefs and focus of the organization. Day to day behavior reflects a merger of the ethics, aspirations, and beliefs of the leaders and how all those attributes are perceived and personalized by the individuals who comprise the employees of the organization. In the best organizations, the aspirations of the leaders are shared by the employees, thus enabling each individual to gain both financially and emotionally. Though one cannot create behaviors, strong vibrant cultures can alter behavioral equilibria toward desired values and behaviors.

The task of creating and advancing a new drug discovery technology and converting that technology to effective drugs requires consistent commitment to innovation and innovators, investment in basic research to understand and advance the technology and, importantly, perseverance through many challenges, disappointments, failures, and outright mistakes. Ionis certainly experienced all those types of events. To persevere, our mission needed to be compelling and clearly articulated, the culture coherent and cohesive.

That goal was achieved at Ionis. I am quite confident that if the culture were not strong, Ionis would have failed or given up long before we were able to create RTD discovery. Our mission was, and is, compelling and I am deeply personally committed to it. The first line in our culture statement is one I have thought about on most days of my career: ‘sick people depend on us’. That simple statement is an extraordinary motivator when coupled to a demanding culture committed to innovation and the practice of rigorous scientific inquiry and the growth of every individual in the organization.

### Setting goals

The first step in leadership is to articulate key goals and concrete plans to achieve them. The overarching aspiration was to create an environment in which patient-centered and focused, innovative, rigorous science and the scientists responsible for the research would thrive. Moreover, those in staff positions such as finance, facilities, human resources must see their jobs as critically important because they support the innovators and innovation. Ideally, each person involved should feel he/she is a key component of the effort to bring better treatments to patients and must experience personal emotional gain. To achieve the desired culture, the following approaches were implemented and maintained throughout the next three decades.

### Establish a scientific organization led by practicing scientists

In most corporate organizations in the pharmaceutical industry, the senior scientific leaders report to business leaders who have demonstrated broad leadership and management skills, but have specific expertise in finance, marketing, or sales. Occasionally, corporate leaders come from a manufacturing or operations background. In some research based pharmaceutical companies and more frequently in biotechnology companies, the senior leader was a scientist, but in most instances, the senior corporate leader will have given up the day-to-day practice of science.

In my experience, it is a rare non-scientist that has sufficient insight into the scientific process to ask the key questions or to demand the key controls. The non-scientists who are capable of doing this make it their business to learn enough of the science over time to become more scientifically sophisticated. It is also rare for non-scientists to be comfortable with the ambiguities of science, i.e. the need to think probabilistically and be comfortable with a qualified answer. In science almost all conclusions are probabilistic statements and qualified by uncertainties. Further, science advances rapidly with new methods being adopted constantly. Given that, unless a scientist continues to be an active participant in the scientific process, it is harder to understand where potential issues with a particular new method may reside.

Another reason to demand that senior scientific leaders continue to lead and publish their own scientific efforts is to avoid obsolescence. A common observation in the industry that I certainly experienced was that scientists tended to become technologically obsolete, yet in academia, senior scientific leaders remained current and relevant far longer and many never became obsolete. I concluded that that was the case because in academia, remaining current, adopting new methods and approaches and publishing were demanded. I therefore established that requirement at Ionis. I was broadly involved in almost all areas of the science, but my small research group focused on my interest to understand the molecular mechanisms by which ASOs and later, siRNAs produce their effects.

I once believed that if I set an example, others would follow. However, I learned that my assumption is typically incorrect. Consequently, it essential to set the expectation that the leaders at all levels in R&D should continue to contribute as individuals to the science of the organization. This is achieved easily by demanding that all the R&D leaders have in their objectives clear, measurable, personal scientific objectives, including publications.

Demanding that scientists at Ionis publish rigorous scientific manuscripts also created collateral value. It assured that Ionis scientists advanced in peer recognition and were, therefore, invited to speak at scientific meetings, contribute scholarly reviews and help establish numerous collaborations with academic leaders. Such interactions enhanced productivity and assured additional layers of interrogation, review, and critiques. Further, our publication history enhanced the ability to recruit scientists at all levels, including postdoctoral trainees. The demand that Ionis scientists publish their work also reinforced the transparency in the organization that is so important to the commitment of individuals to the long-term goals of the company and set us apart from many other companies. The publication of integrated safety databases that summarized safety observations from all non-human primate toxicology studies and all controlled clinical trials for the major chemical classes used at Ionis, is a particularly important example of transparency that I wish other companies would follow (13–16).

Must the CEO remain a practicing scientist to create a rigorous scientific environment? No. However, if the CEO is not a scientist, I am convinced that the senior leader of R&D must be an actively practicing scientist and the CEO must understand the scientific method, be comfortable with the probabilistic nature of science and respect innovation and innovators. Nor do I suggest that implementing what I am suggesting is easy. If a senior leader plans to contribute personally to the science, typically a small group of scientists work directly with him/her. Obviously, this can be organizationally awkward and puts a high premium on the behavior of that leader being pristine and the leader must clearly articulate when he is speaking as a scientist, rather than as CEO. It certainly took time and effort for me to get it right and the Ionis team to be fully comfortable with the atypical organization created.

### Create bespoke human resource and administrative systems to support and reward innovation and innovators

Organizations responsible for innovation are different from traditional manufacturing or commercial organizations. Ideas comprise the key currency of an innovation-centered organization and innovators are the source of success. Conceptual advances and conceptualizers are uniquely valuable and hard to replace. On the other hand, the number of employees supervised is a less important factor in the value an individual may create. To that end, I established systems that were geared to rewards based on the quality of creating ideas and the delivery of results. Individual contributors are much more valuable in an innovation-centered organization than in more traditional corporate tasks. Therefore, special career tracks were created for outstanding, innovative individual contributors that supported them staying in the laboratory yet allowed them to rise to the rank and compensation of a vice president.

In a drug discovery and development organization, the only assets of tangible value to patients are the drugs. Since RTD technology did not exist, innovation was the only route to creating and advancing the technology and the drugs that would derive from the technology. It follows, then, that compensation and other rewards for performance should focus on innovation and innovators and the organization learn to live with eccentricities of many of those individuals. Administrators were taught that their sole responsibility was to facilitate the work that scientists do and the advancement of science. Initially, this was a bit foreign to business and financial contributors who had been in other companies, but over time, most administrative contributors at Ionis came to treasure their contributions to the advances in the technology as they quite correctly took pride in helping the scientists do their work.

### Demonstrate high tolerance of failure but zero tolerance of poor performance

All scientists learn early in their career that experiments can fail, that what seemed like good hypotheses often prove to be incorrect and sometimes major lines of research prove to be impossible with available methods. Those involved in drug discovery and development learn quickly that most drugs fail. So, failure is a part of the everyday life of scientists and a given for those engaged in drug discovery and development. Nevertheless, scientists must take risks if science is to advance.

We worked very hard to avoid being critical of disappointments or blaming or failing to reward solid scientific efforts that simply did not work out. In our organization, when a team performed well, but the drug or the specific project failed, I always began by congratulating the team on their performance and lamenting the fact that the drug or project on which they were working didn’t perform as well as the team. On the other hand, poor performance and sloppy science were dealt with aggressively.

### Create a system to support investigator-initiated research

The great strength of the American scientific process has been that individual investigators have had the latitude to apply for grants in their particular areas of interest, rather than a central authority determining what work should be done. Moreover, most scientists are more excited and successful when they pursue their own ideas rather than interests dictated to them. Effecting such a system is a bit more difficult in a corporate setting because there are well defined corporate goals that define specific areas of interest. However, strategic areas of interest are typically quite broad. In our case, we referred to investigator-initiated work as feasibility studies. Each scientist was given the latitude to pursue his/her specific interests within the context of his/her position, time, and budget. If feasibility was shown, then the investigator could acquire more substantial by presenting the opportunity to senior management. Many of our most successful efforts emerged from this system. A good example is our CNS program. Initially, because of concerns about the safety and breadth of utility of intrathecal dosing, I was skeptical, but the CNS ‘skunkworks’ yielded proof of feasibility and value and became one of our larger and more successful efforts.

I was familiar with the practice in some companies to allow scientists to pursue their own scientific interests during a small fraction of the work day but rejected that as an approach. In a corporate environment, I think it is important that all the innovators be excited about the mission. In our case, it was to create and advance a new technology and use that technology to discover and develop novel medicines. I felt that our mission was broad and intrinsically personally rewarding and wanted every person in the company to be emotionally committed. I also believed that individuals in the company should see everything they did as critical to the mission and that all the science in the company undergo rigorous peer review.

When a feasibility study successfully transitioned to program status, the organization celebrated the success of the leader of the feasibility study and immediately added resources to the leader's team. In contrast ‘personal research’ often is not consistent with the high level organizational objectives and is unlikely to undergo meaningful peer review in the company. Because the feasibility studies are aligned with the mission of the company, the approach is far more likely to be sustainable, even during periods of limited financial flexibility, than research not directly contributory to the mission.

### Maximize transparency

#### Weekly data club

Many organizations aspire to being transparent, but in my experience, a majority of the key activities, meetings and decisions in most corporate organizations are made in private and rarely communicated explicitly to the people who must do the work. Four types of meetings were established at Isis/Ionis, and, in aggregate, they became the heart of the company. All these meetings were open to any person in the company, academic collaborators, consultants and industry partners and, in fact, were always well attended. A corporate wide ‘data club’ meeting has been held weekly for more than three decades. The purpose of this meeting was to celebrate, and peer review the science performed by individual scientists. Typically, there were two 30-min presentations a week. Of course, as the company grew, the attendance at the meetings increased. Nevertheless, they remained relatively relaxed and interactive.

#### Program reviews

Program reviews were more formal reviews of progress in specific programmatic areas, such as medical chemistry, genomics, antisense oligonucleotide screening, molecular mechanisms, pharmacokinetics, toxicology, and various therapeutic programs. These meetings typically lasted 4 h and though they do focus on objectives, they include hours of comprehensive science and opportunities for detailed interrogation of the science, emphasizing again the importance of constant peer review. Once again, these meetings were open to any Ionis employee, consultants, collaborators representatives of industry partners.

Initially there was meaningful reluctance to make these meetings available to all employees and concerns included anxiety about delays, disappointments and negative findings adversely affecting morale and retention of employees. In particular, unease centered on the risk that toxicologic findings might be misunderstood or even misused and perhaps leaked outside the company. Over time, as none of these events occurred and discomfort dissipated. These meetings not only emphasized the importance of the science, but also provided additional opportunities for multi-level peer review, kept employees fully informed and helped all, including non-scientists, to feel they were a part of the progress.

#### Research management and development management committees

These meetings were senior decision-making meetings and I estimate that 90% of the major decisions in the company were made in these forums. As these were decision making meetings, leaders of various efforts presented proposed objectives along with supporting data to the senior leadership. To broaden and sharpen participation, two independent referees for each presentation were tasked with performing more detailed peer review and leading the deliberations. Discussions were typically focused on scientific details or development or commercial issues and were often quite spirited.

These meetings provided a forum to expose key decisions to broadest and most senior peer review and since they were usually attended by >50 non-executive employees, it was an opportunity to see senior leaders making and implementing the decisions that could affect their futures. Although there was initial reluctance among senior leaders to conduct such meetings in public, over time most came to see the value and of the meetings being open and to enjoy the opportunity to discuss critical decisions openly. They also greatly enhanced employee commitment and they proved to be excellent opportunities to teach employees about drug discovery and development.

#### Personal transparency

In addition to establishing various venues in which data, critical decisions, peer review and interrogation of the scientific, medical, ethical, and commercial challenges and issues were discussed in the presence of any individual in the organization who wished to attend, I think that leaders need to personally transparent, both intellectually and emotionally. I worked diligently to personally know as many people in the organization as possible.

Most days when I was in the office, I made at least two trips through the halls, labs, and offices. They were actively purposeful exercises. I spoke to everyone I met and often stopped to chat about the work they were doing and how they and their families were doing. On many occasions, the science being conducted was of interest to me and I sat in the cubicle or office and looked at data with individuals. This built personal bonds and gave me chance to get closer to the raw data and experimental approaches. I also actively listened and wanted to hear lots of chatter and laughter.

Because I like to see the folks in my little research group briefly every day, I was in the lab frequently and that gave me a chance to actually see scientists working and observe the way they planned and conducted experiments. If you want to know what is going on in the lab, be in the lab.

During difficult times, organizations are in the most need of leadership. For every crisis, and we had many, I led all employee meetings. I believe that employees should be told the truth, particularly during crises. They have to make decisions about their lives, and their families and most people need a weekly paycheck. But telling the truth about the cause of the crisis is step one. What must follow immediately is a plan to move forward and frequent updates. During difficult times, I communicated much more frequently and when the crisis was resolved, I shared a revised vision and plan.

#### Task forces

Many times, during our journey, issues occurred that if not understood and resolved could have destroyed the company and the technology. In a company like Ionis the critical issues are related to clinical activities and the solution to the problem derives from scientific studies. In all cases, I formed multidisciplinary task forces and typically led the task force. These focused groups also gave me the opportunity to lead a multidisciplinary team. They force people from different areas of the company to work together under pressure. Thus, they not only helped solve the problem, but created very close bonds between the taskforce members and each other and me.

#### Emphasize the importance of multi-layer peer review and publications

As is obvious from the discussion above, numerous opportunities for detailed peer review and interrogation were built into the fabric of the company. This clearly led to far better and more thorough scientific effort and broadened education of employees as progress in the technology advanced our understanding. Individual leaders were given the latitude to manage their groups as they chose, but most emulated the approach adopted by the senior leaders. I still meet with my small research team focused on molecular mechanisms weekly. Of course, I also meet with the scientists in my group individually as well. Primarily to save preparation time, scientists in my group simply brought their research notebooks, giving me the opportunity to look at the raw data.

Publication of results in peer reviewed journals was required of every scientist at Ionis, including senior leaders and there were specific publication objectives set for each scientist every year. The obligation of scientists to share learnings with others was emphasized and processes that assured that once patents were filed, publications could rapidly be approved for submission were established. This process provided the needed protection of inventions paid for by shareholders while demanding another opportunity for peer review and assuring that our scientists advanced their careers in the broader scientific community.

#### Reward arriving at the ‘true answer, not the desired answer’

To create champions, yet protect against over-zealous championship, all program and project leaders were tasked with arriving at a ‘true’ answer on time and on budget. When the ‘truth’ was the desired outcome, I congratulated the team on their performance and the performance of the drug or specific item in the technology. Teams that arrived at the ‘true’ answer on time and on budget were rewarded equally whether the result was the desired result or a disappointment. Even the failure of drugs must be categorized as a qualified truth that is dependent on the patient population studied and numerous other controllable and uncontrollable variables. Science requires modesty before the challenges of science for all who are willing to learn that lesson.

#### Provide opportunities for scientific advancement based on performance and agnostic to formal training (PhD)

Advanced degrees such as PhD degrees are tickets into faster lanes of career advancement, but once the scientist is recruited, the scientist's work and value are defined by on the job accomplishments. Many excellent scientists do not choose to obtain an advanced degree for many reasons yet are capable of outstanding scientific performance. Consequently, if a non-PhD scientist consistently demonstrated the ability to contribute outstanding scientific results, those individuals at Ionis could ‘earn a ticket to the fast lane’ and be expected to contribute scientifically like a PhD might and be rewarded in a fashion consistent with the quality and quantity of scientific productivity of a scientist with that advanced degree. In fact two of the best examples of the value of this approach worked in my research group and are first authors on many of my publications and grew to have the titles and compensation they earned.

#### Emphasize the value of transient trainees

One approach that enhances productivity and the acquisition of new ideas, techniques and methods in academia is the constant influx of new students and post-doctoral fellows. From day one, the importance of contributing to the training of graduate students and post-doctoral fellows was emphasized and participating as a faculty member in academia encouraged. We also have had a robust post-doctoral training effort that has resulted in many advances and the addition of many outstanding permanent employees. The quality of mentoring was assessed by discussions with fellows, questionnaires, publications, presentations and only mentors who did an excellent job of training were allowed to compete for support for funding of additional post-doctoral positions. Throughout most of my career, I have help active faculty positions and trained a good many graduate students at both Baylor Medical School and the University of Pennsylvania Medical School. At Ionis, we have a number of employees with joint appointments at UCSD, some with primary appointments in Ionis and others with primary appointments at UCSD. I am confident that most perceived barriers are nonsensical and have ignored them.

#### Commit to consistent investment in basic research

Through thick and thin, setbacks and disappointments, we invested in basic research to advance the technology because that was the only way that potential issues could be understood and addressed. Quite a number of times, this meant stepping away from some drugs in development and other desired investment to support basic research. This practice differed dramatically from the conventional wisdom of the time which was embodied by the advice to focus on a single drug.

Another solid commitment was to advance drug discovery programs logically to contiguous areas of interest rather than the disruptive more traditional approach of stochastic terminations of drug discovery programs and initiation of new programs. A good example is the evolution of the cardiovascular program from a limited focus on liver produced lipoprotein cardiovascular risk factors, to new, not yet validated risk factors such as apolipoprotein-CIII and lipoprotein (a). The program then advanced to various targets to treat metabolic disease, including non-alcoholic steatohepatitis (NASH) and from there to resistant hypertension, cardiac arrhythmias, congestive heart failure and renal disease. Disrupting drug discovery research with sudden changes in the ‘strategic direction’ of research, rather than letting programs evolve naturally results in waste and demoralizes scientific teams.

#### Encourage and facilitate a broad network of scientific collaborations

As a company facile in RTD technology, we had numerous ASOs and siRNAs that were excellent research tools for scientists interested in evaluating the roles of various molecular targets in pathways, networks, pathophysiological processes and homeostasis. Scientists were strongly encouraged to collaborate broadly, provide the tools we created and work with outside investigators to assure that the tools were used effectively. Further, the administrative systems needed to establish collaborative relationship sand share various tools and samples were streamlined to make it easy to effect these partnerships. The collaborations yielded a vast array of dividends, include novel ideas, deeper insights into specific therapeutic areas, many excellent manuscripts, and enhanced reputations for Ionis scientists.

#### Commit to retain long service employees

As we were creating a new technology and drug discovery platform, I felt it vital to retain the people who created that knowledge. To that end, we invested substantially in compensation and recognition of long service. For example, one of the highlights every year was an all-employee meeting in which we celebrated long service employees where significant financial awards were associated with long term service. However, I would say that the prominence of the commitment to long service employees and the public recognition were more important that the bonuses. Later, we developed lifetime achievement awards that became quite cherished.

Despite being highly demanding and enduring many disappointments and difficult days, if employees stayed for two to three years, they rarely left voluntarily. For many years our annual voluntary turnover rate was 2–4% compared to an industry average of more than 20%. Of the 30 employees at the founding of Ionis, more than half either still work at the company or have retired after a long career with the company.

## CONCLUSIONS

I established Ionis to achieve a specific mission: to invent, advance and validate a new drug discovery platform from scratch. To have any chance for success, I knew that we would have integrate innovation in many different scientific areas and continue to be highly innovative over decades. I expected disappointments and failures and we certainly experienced many more of the events than I expected. I created the approach and all the administrative approaches with a single objective: facilitate and support innovation and innovators for a decade.

I believed that I had three major assets that needed to be exploited fully. The motivation inherent in the statement ‘sick people depend on us’ is incredibly powerful and I used that every single day to demand more from everyone. The vision was and is extraordinarily broad and compelling. As the largest of dreams in our industry, it provided ample opportunities for people to achieve their goals while contributing to the achievement of the organization's dream. I traded on that every day, especially during hard times. The third asset was the retention of the best scientific and medical talent over the three decades. The low turnover meant that we did not lose knowledge, but as important was the coherence of vision and the confidence in one another at all levels in the organization that derives from overcoming challenge after challenge. Without the full use of all three assets, I am confident that Ionis would have failed.

Just as the approach taken to achieve our goals at Ionis would not be ideal in the pursuit of other missions, this specific bespoke approach is not for everyone. Many individuals, particularly non-scientists, are acutely uncomfortable with peer review. Others are uncomfortable with making difficult judgements in public. Still other prefer a more ‘consensus’ approach to decision making. I made it clear that Ionis was not for everyone and over time, if individuals were to leave the company voluntarily, it typically happened within their first two years of employment, but those who stayed longer than two years typically became long service employees.

In the end, the ethics, quality, and rigor of science are a derivative of ethics and commitment of individual scientists. Leaders of scientific enterprises, whether they are small labs, large labs, research institutes or corporate, for profit, entities can make an enormous difference by committing to and demanding rigorous scientific enquiry and establishing cultures in which such endeavors thrive and are rewarded.
